# Horizontal transmission of heat-evolved microalgal symbionts in adult corals

**DOI:** 10.1093/ismejo/wraf157

**Published:** 2025-07-30

**Authors:** Bede G Johnston, Matthew R Nitschke, Wing Yan Chan, Madeleine J H van Oppen

**Affiliations:** Australian Institute of Marine Science, Townsville, QLD 4810, Australia; School of BioSciences, The University of Melbourne, Parkville, VIC 3010, Australia; Australian Institute of Marine Science, Townsville, QLD 4810, Australia; School of Biological Sciences, Victoria University of Wellington, Wellington 6140, New Zealand; Australian Institute of Marine Science, Townsville, QLD 4810, Australia; Department of Biochemistry and Pharmacology, The University of Melbourne, Parkville, VIC 3010, Australia; Australian Institute of Marine Science, Townsville, QLD 4810, Australia; School of BioSciences, The University of Melbourne, Parkville, VIC 3010, Australia

**Keywords:** coral bleaching, symbiont expulsion, thermotolerance, horizontal transmission, coral reef restoration, experimental evolution

## Abstract

Traditional coral reef restoration methods often fail to consider rising sea-surface temperatures driven by climate change. The introduction of experimentally heat-evolved algal symbionts into corals offers a promising solution by enhancing coral holobiont thermotolerance in a relatively short timeframe. However, the scalability of this approach remains a key challenge. Coral expulsion of viable symbiont cells may provide a passive pathway for upscaling this intervention by facilitating the widespread transmission of heat-evolved symbionts and their physiological benefits across coral reefs. Here, we investigated the expulsion and horizontal transmission dynamics of heat-evolved *Cladocopium proliferum* (strain SS8) in the scleractinian coral *Galaxea fascicularis*. First, we assessed the 24-hour symbiont expulsion dynamics of three colonies of *G. fascicularis* hosting SS8 in addition to homologous symbionts. SS8 was detected in the expelled symbiont community of all colonies, with diel peaks in mitotic index and photochemical efficiency observed at night and the majority of expelled cells appearing morphologically intact. Second, we tested whether expelled SS8 could be acquired by chemically bleached adult *G. fascicularis* fragments in a custom-designed multi-lane raceway experiment. After 55 days of exposure to an SS8-expelling *G. fascicularis* donor, SS8 was detected at background levels (≤0.06%) in 11.1% of recipient fragments (5/45). These findings provide the first empirical evidence that viable, heat-evolved symbionts can be expelled and acquired by bleached adult corals, highlighting a potential natural pathway for the scaling up of this intervention to enhance coral thermal resilience.

## Introduction

Scleractinian corals form the foundation of coral reef ecosystems largely through their symbiotic relationship with photosynthetic dinoflagellates (family: Symbiodiniaceae) [1]. Residing within coral gastrodermal cells, these microalgal symbionts are integral to coral physiology, recycling host metabolic waste products and supplying up to 90% of the coral’s energy requirements through organic nutrient production [2–5]. However, environmental stressors—including elevated sea surface temperatures and excessive light exposure—can destabilize this relationship, leading to mass symbiont loss i.e. coral bleaching [6, 7]. Over the past three decades, climate change has driven an increase in both the frequency and severity of mass bleaching events [8], highlighting the need for innovative strategies to enhance coral thermal resilience [9].

One promising approach involves the use of *ex hospite* laboratory selection to generate Symbiodiniaceae strains with enhanced thermal tolerance [10–12]. Symbiodiniaceae make ideal candidates for this approach due to their relatively short asexual generation time, which can be further contracted in culture, thereby accelerating adaptive evolution [13]. Following the successful development of heat-evolved (HE) *Cladocopium proliferum* strains by Chakravarti, et al. [10], some were shown to significantly improve survival rates and reduce bleaching severity of *Acropora tenuis* larvae and juveniles under elevated temperatures [14, 15]. More recently, the HE strain, *C. proliferum* SS8, was successfully introduced into adult fragments of *Galaxea fascicularis*, conferring thermal tolerance comparable to corals associated with members of the naturally thermally tolerant genus *Durusdinium* [16] without incurring the metabolic trade-offs often experienced with such associations [17, 18].

Despite the demonstrated potential of HE Symbiodiniaceae, several challenges must be addressed before this restoration intervention can be implemented, one of which is identifying pathways that enable the introduction of HE symbionts into wild coral populations at ecologically meaningful levels [12]. This study explores whether the natural phenomena of symbiont expulsion and uptake from the water-column could serve as a mechanism for scaling up the use of HE symbionts to enhance coral thermal bleaching resilience.

Symbiodiniaceae are regularly expelled by their coral hosts via non-lytic exocytosis (vomocytosis) as a means of regulating their population [13, 19, 20], with some corals releasing up to 169 500 symbionts per day per cm^2^ of tissue under ambient conditions [21]. Despite the ionic shock experienced upon release into the marine environment [22], expelled symbionts have been shown to remain physiologically viable under both ambient and stressful temperature conditions [23–26]. This suggests that expelled symbionts have the potential to persist in various environmental reservoirs, including the water column [27], sediment [28], and on macroalgal surfaces [29, 30]. Collectively, these sources form the “symbiont reservoir”—a dynamic pool of free-living Symbiodiniaceae that serves as the primary source of symbionts for the approximately 85% of coral species which must acquire their symbionts de novo each generation (horizontal transmitters) [28, 31, 32]. Beyond early life stages, recent research by Scharfenstein, et al. [33], has demonstrated that adult corals can also incorporate novel, heterologous Symbiodiniaceae from the environment (see also Armstrong, et al. [34], for environmental uptake of homologous symbionts). Together, these findings suggest that expelled symbionts may represent a symbiont source for corals across various life stages.

Given the large-scale occurrence of symbiont expulsion on reefs [26, 35–39] we propose that deploying healthy adult corals hosting HE symbionts has the potential to initiate a cycle of symbiont expulsion, uptake by wild corals, and subsequent re-expulsion—ultimately facilitating a reef-scale introduction of HE symbionts into the symbiont reservoir. For this approach to be viable, however, several key conditions must be met: expelled HE symbionts must be (i) physiologically viable, (ii) capable of at least short-term survival and/or proliferation in the surrounding environment, and (iii) able to be acquired by surrounding corals.

**Figure 1:**
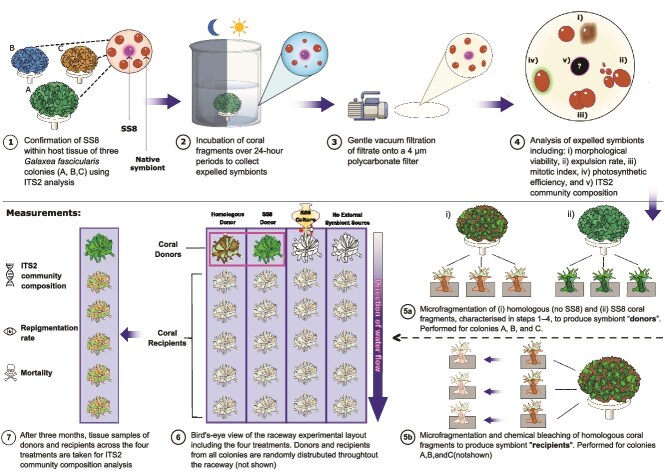
Experimental overview: A schematic diagram depicting the major components of the experimental work. Steps 1–4 show the steps involved in the collection and analysis of expelled symbionts from *G. fascicularis* colonies. All donor fragments, including SS8 and homologous (no SS8) types, were characterized using the same physiological and molecular analyses described in steps 1–4. Steps 5–7 depict the generation of coral donors and recipients, as well as the design and execution of the raceway experiment. To generate coral recipients, homologous *G. fascicularis* fragments (4–5 polyps) from colonies A, B, and C were chemically bleached (supplementary information: Methods S2, S9) and affixed to the Centre of PVC tiles before being randomly distributed across the raceway. The raceway comprised 12 rectangular lanes (455 mm × 24 mm, length × width), separated by 23 mm-high walls. A 5 mm chock (30 mm length × 18 mm height, angled at 31°) was positioned at each lane’s outlet to facilitate water flow. The raceway operated as a non-recirculating system, with FSW flowing continuously through the lanes. Flow rate and temperature were controlled via a SIMATIC WinCC SCADA system (Siemens) at the national SeaSim facility. A flow speed of ~1.3 cm s^−1^, shown previously to permit symbiont acquisition in coral recruits (pers. comm., Dr. Saskia Jurriaans), was maintained. Corals were fed daily with *Artemia nauplii* (0.5 nauplii/ml). Filamentous algae and biofilm were removed from the PVC tiles once per week, and water temperature was maintained at 25.5–26°C. Experimental treatments were as follows: (i) SS8-donor, (ii) homologous donor, (iii) cultured SS8 supplied at densities known to initiate symbiosis (positive control), and (4) no external symbiont source (negative control). To replicate the hydrodynamic conditions present in donor lanes, a coral skeleton of similar size and shape to the live coral donors was placed upstream of the water inlet in both the positive and negative control lanes, providing comparable flow disruption. Each treatment was replicated three times across the 12 lanes of each of the three raceways (n = 9).

This study evaluates the horizontal transmission potential of SS8 symbionts expelled from *G. fascicularis* using a two-phase approach. First, we characterized the expulsion dynamics of three *G. fascicularis* colonies (“donors”), including expulsion rates and symbiont viability over a complete diel cycle. In the second phase, we tested for SS8 uptake in chemically bleached *G. fascicularis* fragments (“recipients”) using a multi-lane experimental raceway system (Fig. 1). By assessing both the viability and transmissibility of expelled SS8 symbionts, this study offers new insights into the potential for coral hosts to act as vectors in the large-scale deployment of healthy, actively dividing, thermally tolerant symbionts into reef ecosystems.

## Materials and methods

### Collection and maintenance of *G. fascicularis*

Three colonies of *G. fascicularis* (genotypes A, B, and C) were collected from Davies Reef in the central region of the Great Barrier Reef (GBRMPA permit: G12/35236.1) and acclimated for two weeks in the National Sea Simulator at the Australian Institute of Marine Science. Each colony was fragmented into 4–5 polyp pieces and affixed to aragonite plugs (Frag Plugs Aragonite Large; OW100LCFP, Aquasonic, Wauchope, Australia) using Gorilla Super Glue. All experimental fragments used in this study—including SS8 donors, homologous (non-bleached) donors, and chemically bleached recipients—were generated from these three source colonies. Fragments were left to recover in 2 μm filtered seawater (FSW) for four weeks in 50 L experimental tanks. See Supplementary Information: Methods S1 for details on coral husbandry.

### Heat-evolved symbiont (SS8)

The heat-evolved symbiont used here was *C. proliferum* (SCF 055.01.08; referred to as SS8 as per [14–16]). The Internal Transcribed Spacer 2 (ITS2) profile of the SS8 inoculum is C1-C1b-C42.2-C1bh-C1c-C1br-C1cb-C1ge-C1w-C3ju-C72k-C1al, C42ca-C3sa-C42ao-C1jx-C1cu [33].

### Generation of SS8–*G. fascicularis* donors

SS8–*G. fascicularis* donors were generated via chemical bleaching and inoculation with cultured SS8 (for details on chemical bleaching and inoculation see Supplementary Information: Methods S2). Once bleached, donors were inoculated with cultured SS8 (final cell density: 1000 cells ml^−1^) during its peak motile period (~1 hour after culture incubator lights turn on) over a 7-day period and allowed to repigment for 8 weeks. Coral recovery was monitored via image analysis of colour and dark-adapted maximum quantum yield of photosystem II (*F_v_/F_m_*) (Supplementary Information: Methods S3). ITS2 community composition analysis confirmed the presence of SS8 defining intragenomic variants (DIVs) in donors (details provided below) and absence of SS8 DIVs in controls (naïve *G. fascicularis* fragments, not chemically bleached, herein referred to as homologous donors).

### Collection and analysis of expelled symbionts from *G. fascicularis*

#### Expelled symbiont collection

Expelled symbionts were collected by incubating individual *G. fascicularis* fragments in 1 L plastic beakers (700 ml of FSW, ~26°C with continuous gentle aeration). One fragment per colony was analysed. In addition to SS8-donors, homologous donors were also analysed. Given the influence of photoperiod on coral and symbiont physiology [40, 41], six 4-hour incubations were conducted over a 24-hour cycle: morning (08:00–12:00), midday (12:00–16:00), dusk (16:00–20:00), evening (20:00–00:00), night (00:00–04:00), and dawn (04:00–08:00). Lighting was programmed to turn on at 06:30 and off at 16:30, with a gradual two-hour ramp-up and ramp-down period (see Supplementary Information Fig. S1). After each incubation, corals were carefully rinsed to remove surface mucus and transferred to fresh beakers. The incubation seawater was gently stirred to dislodge any adhered symbionts, then gravity-filtered through a 63 μm mesh to remove larger particulates. Expelled symbionts were subsequently collected via vacuum filtration onto 4 μm polycarbonate filters (Isopore membrane, TMTP09030, Millipore, Billerica, MA, USA) using a Millivac-Mini Vacuum Pump (230 V). The time between symbiont collection onto filters and downstream analysis was minimized to 20–30 minutes. All procedures were repeated on three separate days, with a minimum interval of 5 days between repetitions, using identical methodologies.

#### Symbiont expulsion rate

Expulsion rates (cells expelled cm^−2^ hr^−1^) were determined through manual cell counting using a fluorescence microscope (Zeiss AxioVert 200 M). Half of the 4 μm polycarbonate filter was used for expulsion rate calculations, determination of mitotic index (MI), and morphology assessments, whereas the other half was reserved for measuring *F_v_/F_m_* and ITS2 community composition (methodology detailed below). Filters were hydrated with 20 μl FSW before imaging under 20X magnification. Avoiding image duplication, 10–15 images were captured per filter, depending on symbiont density, averaged, and then extrapolated to total filter surface area (4.9 cm^2^). Counts were normalized to the tissue surface area of the corresponding *G. fascicularis* donor, measured using 3D photogrammetry as described by Figueira et al. (2015) [42] (Supplementary Methods S4).

#### Morphological viability, mitotic index, and photochemical efficiency

Following imaging, expelled symbionts were carefully washed from the filter into a 1.5 ml Eppendorf tube using pipetting, resuspended in 1 ml of 2% glutaraldehyde in FSW and stored at 4°C in the dark until further analysis. Prior to observation, symbionts were pelleted by centrifuging at 1000 *×* g for five minutes. The supernatant– confirmed via light microscopy (Zeiss AxioVert 200 M) to be free of residual cells—was discarded, and the pellet was resuspended in 250 μl of FSW. To obtain *in hospite* cells, a small portion of coral tissue was gently scraped and suctioned using a plastic Pasteur pipette between 20:00–00:00. *In hospite* cells were stored and prepared using the same methodology as for expelled cells.

**Figure 2:**
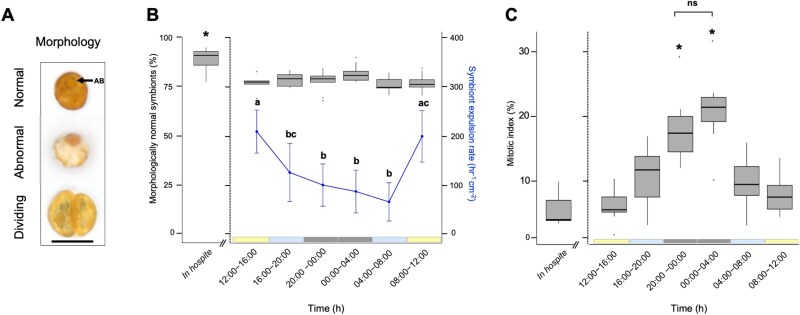
Physiological characteristics of *in hospite* and expelled Symbiodiniaceae from *Galaxea fascicularis*. (A) Representative light microscopy images showing morphological states of symbionts: normal, abnormal, and dividing. Scale bar = 10 μm. Arrow pointing from AB indicates the accumulation body. (B) Rate of Symbiodiniaceae expulsion (total number of cells of all three morphological categories expelled hr^−1^ cm^−2^ of coral surface area) measured over six consecutive 4-hour incubations (right Y axis). Percentage of morphologically normal *in hospite* and expelled cells (left Y axis) across the same 24-hour interval. Dividing cells were also classified as normal. (C) *In hospite* and expelled symbiont mitotic index (proportion of dividing cells) measured over six consecutive four-hour incubations. Line graph data represent mean ± 1 standard deviation. Statistical significance is denoted by different letters (a, b, c), where groups sharing the same letter are not significantly different from one another. “ns” indicates a non-significant difference between the compared groups. All measurements were repeated over three separate days.

Morphological viability was assessed following [35]. Briefly, normal symbionts were identified as brown, circular cells with an intact cell wall, reticulated chloroplast, and a large accumulation body (see Fig. 2A for representative images). Abnormal cells were irregular in shape, lacked a cell wall, and/or had diameters less than 50% of healthy cells. The number of normal versus abnormal symbionts (n = 50–120) was recorded as a percentage of the total population. Mitotic index was calculated as the proportion of cells displaying a doublet morphology or clear visible division [13]. Cells classified as dividing were counted towards the normal morphology category.

To assess photochemical efficiency, the remaining half of the filter was used to measure the *F_v_/F_m_* of expelled symbionts using a Walz Microscopy Pulse-Amplitude Modulated fluorometer (Microscopy iPAM; Walz GmbH, Effeltrich, Germany; see Supplementary Information: Methods S5 for full details). Measurements were performed on ~120 individual cells per *G. fascicularis* colony per timepoint at 20× magnification, with fluorescence excited using a 470 nm blue Zeiss LED module. Data were collected using ImagingWin software (v2.32 FW Multi RGB). Outliers were removed based on a Z-score threshold of one (Supplementary Information: Methods S6). Following measurements, filters were stored at −80°C for subsequent ITS2 community composition analysis.

### DNA extraction, sequencing, and SymPortal analysis

Details on DNA extraction, PCR amplification and library preparation are provided in Supplementary Information: Methods S6. Sequencing of the ITS2 region of Symbiodiniaceae ribosomal DNA was conducted on a MiSeq System (Illumina) using a v3 kit (2 × 300 bp) at the Walter and Eliza Hall Institute, Melbourne. Demultiplexed raw sequences were submitted to SymPortal for ITS2 sequence analysis (Supplementary Information: Methods S7). The presence and abundance of recurring ITS2 sequences across samples are identified by SymPortal as “ITS2 type profiles”, which are representative of putative Symbiodiniaceae taxa. Each of the sequences in the recurring set (the profile) is referred to as a DIV. Due to the relatively low abundance of the SS8 inoculum in experimental corals, the presence of SS8 was primarily indicated by the most dominant DIVs. Sequencing data was downloaded from SymPortal and analysed in R version 4.2.0 (R Core Team, 2022). DNA sequencing and ITS2 analysis were performed on day one of incubations only. Resulting proportions are reported as relative abundances of ITS2 gene copies (hereafter referred to as relative abundance), to reflect that these values quantify the relative frequency of multicopy ITS2 rDNA sequences within the sequencing dataset, rather than the absolute or relative abundance of symbiont cells.

### Raceway design and validation

To examine horizontal symbiont transmission from donor to recipient, a custom raceway was designed using CAD software (AutoCAD Inventor, Autodesk Inc., San Rafael, CA, USA), and constructed from polymethylmethacrylate panels using a computerized numerical control milling machine (Mazak VCN 530, Yamazaki Mazak, Oguchi, Japan). The design was adapted from a multi-lane swimming chamber used to assess fish swimming performance [43]. For full details on raceway specifications, experimental setup, and flow speed validation see Fig. 1; Supplementary Information: Methods S8, S10; and Fig. S2, S3, and S4).

### Symbiont culturing and dosing

*C. proliferum* SS8 cultures were maintained at 27°C under a light intensity of 40–70 μE m^−2^ s^−1^ following a 12:12-hour light–dark cycle in 1% IMK culture medium (Nihon Pharmaceutical Co.). Cultures were refreshed every four weeks, replacing >50% of the medium with fresh IMK. Further details on the cone and peristaltic pump setup are provided in Supplementary Information: Methods S10. The pump delivery system was configured to continuously deliver ~10 000 SS8 cells per ml into each lane every minute, for 6 hours per day.

### Survival and repigmentation

Survival and repigmentation of *G. fascicularis* recipients and donors were monitored throughout the three-month experimental period. Repigmentation was assessed biweekly via close-up photographs taken within the lanes to minimize disturbance. Corals were imaged at seven timepoints over the 55-day period using an OM SYSTEM Tough TG-7 Digital Camera. Recipient pigmentation was then scored against the Coral Health Chart [44]. The change in pigmentation over time was calculated by subtracting the recipient’s colour score upon placement into the raceway (initial timepoint) from its colour score at each subsequent timepoint.

### Statistical analysis

All statistical analyses were conducted in R version 4.2.0 (R Core Team, 2022). To assess differences in physiological response variables (e.g. morphology percentage, mitotic index, and *F_v_*/*F_m_*), we used generalized linear mixed models (GLMMs) fitted with the glmmTMB package. GLMMs were used to account for the hierarchical structure of the data, with random intercepts for measurement day (i.e. experimental run) and host colony identity (A, B, C) to capture variation across independent replicates and genotypic backgrounds, respectively. Each response variable was modelled separately using a Gaussian error structure. The fixed effect Timepoint included seven levels, representing six post-expulsion intervals and an additional *in hospite* level. Pairwise comparisons of estimated marginal means were conducted using the emmeans package with Tukey correction for multiple testing. The general model structure was:

The general model structure was:


$$ Physiological\ response\sim Timepoint+\left(1\ |\ Day\right)+\left(1\ |\ Colony\right) $$

For analyses of *G. fascicularis* recipient repigmentation rates, linear mixed-effects models (LMMs) were used to account for repeated measures within individual coral fragments. To improve normality, relative colour change values were positive shifted and log-transformed after shifting values to ensure all were positive and above 0. Model assumptions were checked using diagnostic plots and residual analyses. Significance thresholds were set at α = 0.05.

## Results

### Symbiont expulsion rate

Symbiont expulsion rate varied significantly across the six incubation timepoints (Fig. 2B; Fig. S5; Table S1). Expulsion rates were highest at 12:00–16:00 h (214.3 ± 44.8 cells cm^2^ h^−1^) and 08:00–12:00 h (196.3 ± 69.0 cells cm^2^ h^−1^), coinciding with peak lux levels (see Fig. S1 for lux levels over time). In contrast, expulsion rates were lowest during the two nighttime incubations, reaching 85.3 ± 43.8 cells cm^2^ h^−1^ at 00:00–04:00 h and 64.1 ± 39.0 cells cm^2^ h^−1^ at 04:00–08:00 h. For inter-colony trends across all physiological measurements see Fig. S6–S12.

### Morphological viability

The proportion of morphologically normal to abnormal expelled symbionts remained consistent over the six incubation timepoints (Fig. 2B; Fig. S7; Table S2). On average, normal cells comprised 77.6% ± 4.5% of the total expelled populations. *In hospite* populations exhibited significantly higher proportions of normal cells (87.1% ± 6.7%).

### Mitotic index

The mitotic index (MI) of expelled symbionts followed a diel cycle (Fig. 2C), peaking at 21.1% ± 5.7% during the second nighttime incubation (00:00–04:00) before decreasing to its lowest value of 6.1% ± 2.4% during the second daytime incubation (12:00–16:00). MI was significantly elevated during both nighttime incubations compared to all other timepoints with nighttime values ~3.5 × higher than those recorded during the day. The MI of *in hospite* symbionts was the lowest among all measured conditions, averaging 5.8% ± 2.6% (Fig. 2C; Table S3), however, this difference was not statistically significant. Overall, expelled cells from all colonies followed a similar diel pattern (Fig. S10).

### PSII maximum quantum yield (*F_v_/F_m_*)

**Figure 3:**
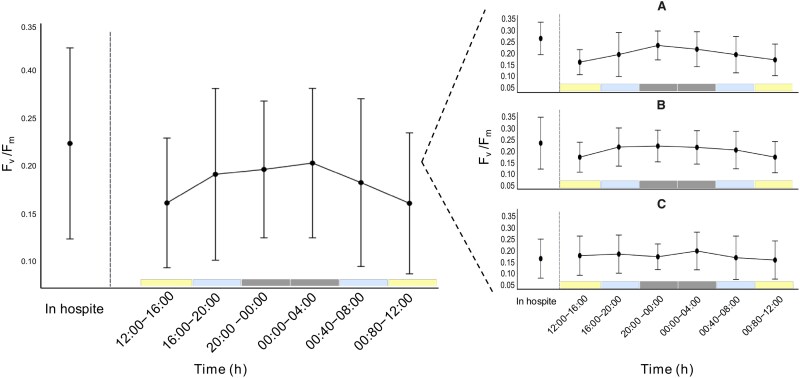
Photochemical efficiency (*F_v_ /F_m_*) of *in hospite* and expelled Symbiodiniaceae from *Galaxea fascicularis*. (A) Maximum quantum yield of Photosystem II (*F_v_ /F_m_*) for *in hospite* and expelled symbionts from *G. fascicularis* over six consecutive 4-hour incubations. (B) Colony-specific *F_v_ /F_m_* data for *in hospite* and expelled symbionts over the same incubation periods. All colonies exhibited a similar diel trend, with *F_v_/F_m_* inversely related to light levels, however, this relationship was strongest in colony A (Fig. S12B). The *F_v_/F_m_* of expelled symbionts from colony C was significantly lower than those from colonies A and B at each respective timepoint, except during the second daytime incubation (12:00–16:00 h). The *F_v_/F_m_* values of *in hospite* symbionts from colony C were also low (0.146 ± 0.086) compared to colony A (0.263 ± 0.071) and colony B (0.228 ± 0.114). When averaged across all timepoints, *in hospite* cells from colonies A and B exhibited 1.5x higher *F_v_/F_m_* than their expelled counterparts (0.273 vs. 0.188 for A; 0.312 vs. 0.195 for B). In contrast, no significant differences between *in hospite* and expelled populations were observed for colony C. Post-hoc statistical significance is denoted by different letters (a, b, c, d), where groups sharing the same letter are not significantly different from one another. Data are shown as mean ± 1 standard deviation. All measurements were repeated over three separate days.

The *F_v_/F_m_* of expelled cells followed a diel cycle, with the lowest values recorded during the two daytime incubations (0.16 ± 0.07 and 0.16 ± 0.07 for 08:00–12:00 and 12:00–16:00, respectively) and the highest values observed at night (0.20 ± 0.07 and 0.20 ± 0.08 for 20:00–00:00 and 00:00–04:00, respectively) (Fig. 3; Table S4). Intermediate *F_v_/F_m_* values were recorded during dusk (16:00–20:00 h) and dawn (04:00–08:00 h), aligning with transitional light phases. *In hospite* symbionts exhibited a significantly higher *F_v_/F_m_* compared to expelled cells across all timepoints, except during the second nighttime incubation (00:00–04:00).

### *In hospite* and expelled Symbiodiniaceae community (ITS2) profiles

**Figure 4:**
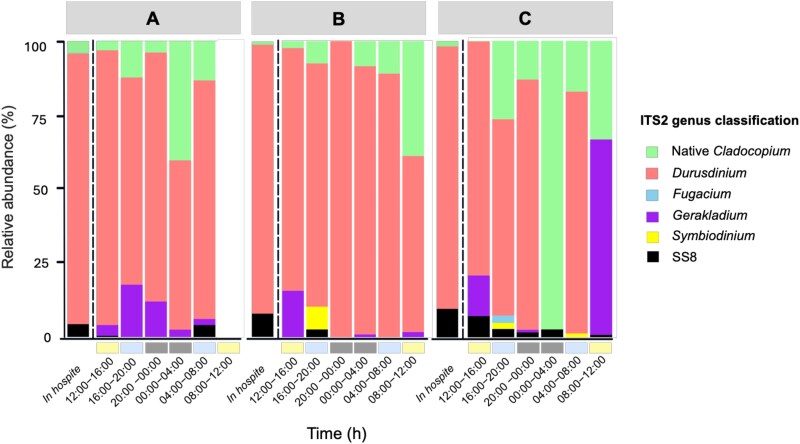
ITS2 community composition of *in hospite* and expelled Symbiodiniaceae genera. *In hospite* and expelled Symbiodiniaceae community profiles from the three *G. fascicularis* colonies (A, B, C) collected over six consecutive 4-hour incubations. Each bar represents expelled or *in hospite* symbionts collected at a specific timepoint with each individual bar representing the proportion DIVs assigned to a specific genus of native symbionts or SS8 relative to the total abundance of sequences. Empty bar indicates sample that did not pass quality control. Expelled symbiont communities displayed high variability across colonies and incubation timepoints. Compared to *in hospite* communities, expelled populations contained higher proportions of native *Cladocopium*, ranging from 3.2%–40% (A), 0%–38.9% (B), and 0.21%–97.3% (C). This increase was accompanied by a corresponding decrease in *Durusdinium*. A further disparity between *in hospite* and expelled profiles was highlighted by the detection of symbionts in expelled communities that were not present in their respective donors. *Symbiodinium* was identified in expelled communities from colony B (7.7%) and colony C (1.3% – 2%), with *Fugacium* additionally detected in colony C (2.5%). *Gerakladium* was consistently detected at varying proportions across all colonies, ranging from 2.1% to 17.7% (A), 0.92%–15.7% (B), and 0.89%–66% (C).

The *in hospite* Symbiodiniaceae community of all donors contained SS8 at low abundances: 4.4% (A); 7.9% (B); and 9.8% (C). Across all donors, *Durusdinium* was the dominant genus making up 91.5% (A), 91% (B), and 88.6% (C) of the total symbiont community (Fig. 4). Native *Cladocopium* was also present in minor proportions, contributing 4.1% (A), 1.1% (B), and 1.6% (C). SS8 was detected in the expelled communities of all colonies, though its relative abundance varied across colonies and timepoints (Fig. 4). In colony A, SS8 was observed at two timepoints, reaching 0.43% at 12:00–16:00 h and peaking at 4.0% at 04:00–08:00 h. In colony B, SS8 was detected at 2.61% only at a single timepoint (16:00–20:00 h). In contrast, colony C contained expelled SS8 across five out of six timepoints, with abundances ranging from 0.79% to 7.13%. In all cases, SS8 was expelled at lower proportions than found in its respective donor colony (see Fig. S14 for ITS2 DIV and type profile symbiont community composition).

### Symbiont repigmentation and mortality in raceways

**Figure 5:**
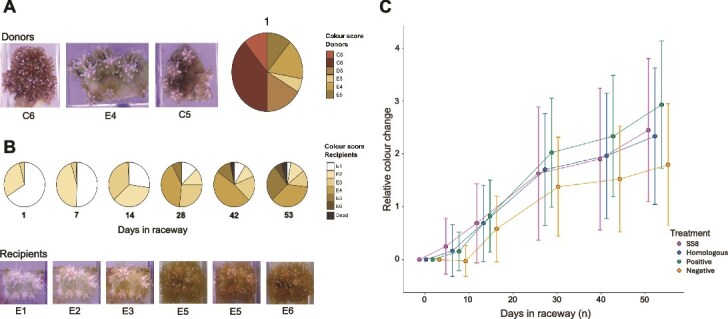
Repigmentation trajectories of raceway recipients over time. (A) Pie chart distributions of donor coral colour scores at the start of the raceway experiment, recorded using the coral health chart [44]. Representative images from donor colonies (C6, E4, C5) illustrate initial pigmentation of colonies A, B, and C respectively. Colour scores showed little change over the course of the experiment. (B) Pie chart distributions of recipient coral colour scores and mortality throughout the raceway experimental period. Representative images of a single recipient showing progressive repigmentation over the experiment. (C) Relative colour change of recipient corals over time compared to their initial T0 score, across all treatments. Data are presented as mean ± 1 standard deviation.

Colour scores of recipient coral fragments increased progressively over time, reaching levels comparable to or slightly below those of donors by the end of the experiment (Fig. 5A, B). Coral mortality was first observed after 42 days in the raceway, and by the conclusion of the experiment (55 days), nine out of 180 recipients (5%) had died, all of which belonged to colony C (Fig. 5B; Table S5). All corals showed significant increases in pigmentation over time (*P* < .001) (Fig. 5C). Although the negative control treatment showed generally lower pigmentation rates, the effect of treatment over time was not statistically significant (*P* = .093). Repigmentation rates varied significantly between colonies (colony:time interaction, *P* < .001) (Fig. S14; Table S6).

### Expelled symbiont acquisition

**Figure 6:**
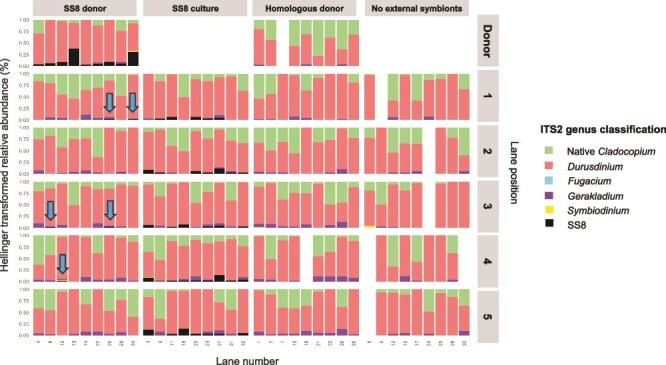
Hellinger transformed ITS2 Symbiodiniaceae community composition of donors and recipients in the raceway experiment. Bars represent the Symbiodiniaceae community profiles of donor and recipient corals across the four experimental treatments: SS8 donor, SS8 culture (positive control), homologous donor, and no external symbionts (negative control). Each bar corresponds to an individual recipient sampled at the end of the experiment, with sections indicating the proportion of DIVs assigned to a specific genus of native symbionts or SS8 relative to the total sequence abundance. Empty bar indicates sample that did not pass quality control. The right axis denotes recipient positions within their respective lanes. A square root transformation was applied to improve the visibility of background symbionts. Arrows indicate instances of successful SS8 transmission within the SS8 donor treatment.

After 55 days of being reared alongside an SS8 *G. fascicularis* donor, SS8 was detected in five out of 45 recipients (11.1%) (Fig. 6; Fig. S15). However, it was detected at very low sequence reads (11–19), never exceeding 0.06% of the total *Symbiodiniaceae* community, classifying it as a background symbiont [45, 46]. Specifically, SS8 accounted for 0.029%, 0.029%, 0.033%, 0.047%, and 0.06% of the symbiont community in these five recipients. In addition to intra-colony horizontal transmission, SS8 was also transmitted between colonies; a donor from colony B transferred SS8 to recipients from colonies A and C, whereas SS8 symbionts expelled from donor colony C were acquired by a recipient from colony B. One sample from the homologous donor treatment was removed from analysis due to the unexpected detection of an SS8 DIV *in hospite* (data not shown). SS8 was not detected in this sample prior to the experiment, nor in the other two micro fragments from the same colony. SS8 was also absent from all recipients reared alongside this donor. Given these factors, the sample was considered contaminated during DNA extraction and excluded from further analysis.

Recipients in the SS8 culture treatment (positive control) had the highest uptake of SS8, with acquisition detected in 34 out of 45 recipients (75.6%). SS8 sequence reads in these recipient fragments ranged from 12 to 808 (average 150 reads), comprising between 0.048% and 3.7% of the total symbiont community (averaged 0.6%). Although SS8 was detected relatively equally across the three colonies (10 recipients from colony A, 14 from colony B, and 10 from colony C), the proportion of SS8 acquired differed significantly (ANOVA: F_2,31_ = 3.528, *P* = .042, Table S7). Specifically, SS8 accounted for an average of 1.2% of the total symbiont community in colony C recipients, in contrast to 0.49% in colony A and 0.26% in colony B (Fig. S15). Post-hoc Tukey comparisons revealed that colony C acquired significantly more SS8 than colony B (*P* = .036), whereas no significant differences were detected between colonies A and B or between colonies A and C. No evidence of SS8 acquisition was detected in recipients reared alongside homologous *G. fascicularis* donors or in those from the negative control.

## Discussion

### HE symbionts are expelled by their coral hosts

For corals to act as effective vectors in the large-scale deployment of SS8 into the symbiont reservoir and, ultimately, wild coral populations, they must be capable of expelling SS8 into the external environment. Our study provides empirical evidence of SS8 expulsion from *G. fascicularis*, confirming that SS8 cells can be naturally released into the water-column through expulsion processes. Although SS8 was found at relatively low proportions in the expelled symbiont communities (<6%), this is unsurprising given its low abundance within the donor colonies (4.4%–9.8%). In all cases, the proportion of expelled SS8 never exceeded the *in hospite* levels of its respective donor. This suggests that beyond expulsion, *G. fascicularis* may regulate SS8 populations through various pre-mitotic mechanisms [47–51]. Alternatively, SS8 may exhibit a slower *in hospite* division rate relative to other symbionts [52] or is preferentially retained by the host.

### Expelled symbionts are physiologically viable

Once expelled, SS8 must remain physiologically viable and capable of establishing new symbioses. A key factor in this process is the structural integrity of expelled cells. Although many corals primarily expel degraded or partially digested symbionts [5, 35, 53–56], our findings showed that over 73% of Symbiodiniaceae expelled from *G. fascicularis* exhibited an intact morphology, with this proportion remaining stable across the diel cycle. This is consistent with previous studies on *G. fascicularis* [57] and *Acropora millepora* [58] where 74–76% of expelled symbionts were reported as morphologically normal (however, see Bhagooli and Hidaka [59]).

A cell’s ability to divide and proliferate is another key indicator of its physiological viability. In this study, relatively high proportions of dividing cells (>6% up to 22%) were consistently observed across the diel cycle, indicating that a substantial fraction of symbionts remain physiologically active upon expulsion. Among the expelled populations, cells released overnight exhibited MI values nearly twice as high as those expelled during the day (Fig. 3; Table S3), consistent with the well-documented photoperiodic regulation of Symbiodiniaceae, in which cytokinesis predominantly occurs in darkness [40, 60, 61]. Furthermore, *in hospite* cells, which were sampled between 20:00 and 00:00, had a significantly lower MI compared to expelled cells at the same timepoint (5.8% ± 2.6 vs. 18.2% ± 5.2). A similar pattern has been observed in other cnidarians [36, 50, 52] with strong evidence suggesting some hosts preferentially expel actively dividing cells as a mechanism of population regulation [52, 59] (however, see [59]). These elevated nighttime cell division rates may also explain the daytime peaks in symbiont expulsion rate (Fig. 2B), with the host attempting to offset overnight increases in Symbiodiniaceae population. In order to determine whether these dividing cells include SS8, future research should conduct single-cell genotyping.

High division rates among expelled symbionts also suggest their potential to establish self-sustaining populations within the symbiont reservoir. Supporting this, Nitschke, et al. [28] demonstrated that expelled symbionts can accumulate in reef sediments. Coral recruits reared near such symbiont-containing sediment exhibited higher colonisation rates compared to those reared in seawater alone, likely due to passive sediment resuspension and symbiont ingestion, or active infection while in a motile form—the primary dispersal and infectious stage of Symbiodiniaceae [62–65]. Although motile cells were not directly observed here, Yeager, et al. [21] recently confirmed their presence among expelled populations from multiple coral species. In addition to short-range dispersal via motility [62, 63, 66–68], expelled symbionts have the potential to be dispersed over long distances through passive oceanic transport [69], ingestion and excretion by corallivorous fish [70], and/or vectoring via coral larvae [71]. To better understand the dispersal dynamics of SS8, future research should investigate the persistence of expelled SS8 cells in the water column, sediment, and other benthic microhabitats such as macroalgae and turf algae [30], alongside motility dynamics and reef connectivity [72].

The *F_v_/F_m_* of expelled cells, although low, did exhibit a distinct diel pattern, with significantly higher values observed at night and lower values during the day. This rhythmicity, consistent with previous findings for cells *in hospite* [73], suggests these expelled cells retain the capacity for dynamic, reversible downregulation of photochemistry—a metabolically demanding process indicative of physiological viability [74].

### Expelled heat-evolved symbionts can be acquired by adult conspecific corals

Once expelled into the external environment, free-living symbionts must overcome several biological and ecological barriers to successfully colonize a new host [75–78]. These include: (1) encountering a suitable coral host, either passively or actively [65, 67, 70, 79], (2) navigating the host’s innate immune response [80], and (3) rapidly establishing an intracellular niche via metabolite translocation [80, 81] (however, see Jinkerson et al. [82]).

The results presented here provide empirical evidence of successful horizontal transmission of HE symbionts between adult corals. Overall, SS8 was detected in 11.1% of the recipient population (five out of 45), confirming that at least a portion of expelled SS8 cells overcame these barriers. Despite successful acquisition, SS8 relative abundance *in hospite* remained extremely low (average 0.04%). However, given that SS8 acquisition/proliferation has been shown to occur slowly, even under conditions of direct inoculation with cultures at higher densities (~10 000 cells ml^−1^), such low proportions within the relatively short experimental timeframe are not unexpected (see Chan, et al. [16] where proportions of *in hospite* SS8 increased over time). Compared to those densities, the estimated number of SS8 cells in the water column in this experiment would have been orders of magnitude lower with peak expulsion rates involving approximately four symbionts expelled per minute per cm^−2^ of donor tissue (Fig. 2B). Despite such low proportions *in hospite*, background symbionts have been shown to play a disproportionately large role in shaping post-recovery symbiont communities [83–85]. For instance, Buzzoni, et al. [84] showed that *Durusdinium* levels as low as 0.27% strongly predicted post-bleaching community shifts often leading to *Durusdinium*-dominated or entirely exclusive symbioses. Given the thermal tolerance conferred by SS8, future studies should assess whether a similar pattern is followed, particularly in response to thermal stress which may enhance its competitive advantage.

For widespread uptake of expelled HE symbiont cells in natural reef settings among hosts, successful inter-genotype and (considering the generalist nature of *C. proliferum*) inter-species transmission will be essential [12]. One potential barrier to this process is the thin (~0.5–1 μm) layer of host-cell membrane and cytosol (symbiosome) that often encloses expelled cells [20]. Retained host material may impede symbiont acquisition by masking key algal surface-associated cues or triggering the coral’s innate immune system thus reinforcing host specificity [86]. Although the genetic relatedness of donor and recipient colonies was not directly assessed, distinct differences in polyp morphology, size, and pigmentation suggest that they were genetically distinct. Here, three out of the five instances of successful inter-colony SS8 transmission occurred: (i) from a colony B donor to recipients from colonies A and C, and (ii) from a colony C donor to a colony B recipient. These findings suggest that remnant host signatures are sparse, lost rapidly and/or may not hamper early symbiosis establishment. Similar results were reported by Rodriguez-Lanetty, et al. [87], who found no difference in infection success regardless of the host source, concluding that symbiosis establishment is primarily dependent on the intrinsic properties of the algae rather than signatures from their previous host. Alternatively, those symbionts successfully acquired by a new host may have been expelled without a symbiosome as observed in Ladrière, et al. [88]. Overall, these findings, combined with the well documented broad host range of *C. proliferum,* including Great Barrier Reef *Galaxea* spp*.* [89], indicate that expelled SS8 cells are not inherently restricted to their original host and may be acquired across a broad range of species.

### Utility of the raceway experimental design

Overall, the controlled raceway environment proved a suitable system for testing horizontal transmission dynamics as evidenced by several key findings. Recipients in the positive culture treatment, exposed to high densities of free-living SS8, exhibited the highest acquisition rates, demonstrating that conditions in the raceway—including light and flow speed and direction—permitted effective uptake of SS8 cells when readily available. In contrast, SS8 was not detected in recipients reared alongside homologous donors or in the negative control treatment, indicating that no detectable contamination between lanes occurred during the experiment. Repigmentation among recipients followed a complex pattern, with a marginally significant three-way interaction between treatment, time, and colony (*P* = .087). Rather than reflecting a simple treatment effect, this suggests that colour recovery varied both temporally and among donor colonies. Although this interaction did not meet the conventional threshold for statistical significance (*P* < .05), it indicates that factors beyond direct symbiont acquisition—such as increased symbiont digestion, host-derived signalling, microbial interactions, or elevated nutrient and metabolite availability—may have contributed to repigmentation within the raceway. This highlights the raceway’s potential to capture subtle, biologically relevant processes beyond the direct transfer of symbionts.

### Conclusions and recommendations

This study demonstrates that viable HE symbionts can be expelled by donor corals and subsequently acquired by adult conspecifics. Although interspecies transmission remains to be tested, the broad host range of *C. proliferum* suggests it is possible [87]. Corals hosting HE Symbiodiniaceae could be strategically deployed to thermally stressed reefs where expelled symbionts may enter the environmental reservoir and, in turn, be acquired by surrounding corals to accelerate recovery. This approach could also be extended to healthy or vulnerable reefs to proactively enhance thermal resilience under future climate scenarios. Overall, these findings highlight the potential for corals to act as biological vectors for HE symbionts and underscore the need to better understand factors influencing their environmental persistence, acquisition, and proliferation. A deeper understanding of symbiont connectivity between reefs could further support targeted deployments, maximising the spread and long-term establishment of HE symbionts to support reef-scale thermal adaptation.

## Supplementary Material

Supplementary_information_Revised_wraf157

## Data Availability

Raw sequences of the ITS2 Symbiodiniaceae datasets are available in GenBank (BioSample accession: SAMN53512600 - SAMN53512653; SAMN53501939 - SAMN53502142; BioProject ID: PRJNA1371389).
