# Correction to: Horizontal transmission of heat-evolved microalgal symbionts in adult corals

**DOI:** 10.1093/ismejo/wrag027

**Published:** 2026-02-21

This is a correction to: Bede G Johnston, Matthew R Nitschke, Wing Yan Chan, Madeleine J H van Oppen, Horizontal transmission of heat-evolved microalgal symbionts in adult corals, *The ISME Journal*, Volume 19, Issue 1, January 2025, https://doi.org/10.1093/ismejo/wraf157

The **Data availability** statement has been emended to read: “Raw sequences of the ITS2 Symbiodiniaceae datasets are available in GenBank (BioSample accession: SAMN53512600 -SAMN53512653; SAMN53501939 - SAMN53502142; BioProject ID: PRJNA1371389).” instead of: “All datasets and analysis scripts associated with this study will be made publicly available upon publication at github.com/XXXX.”

