# Supplementary material for: Horizontal transmission of heat-evolved microalgal symbionts in adult corals
Source: ISME J. 2025 Jul 30;19(1):wraf157. doi: 10.1093/ismejo/wraf157 (PMC12596636; doi:10.1093/ismejo/wraf157)
Supplement: Supplementary_information_Revised_wraf157 [file supplementary_information_revised_wraf157.docx]

**Supplementary Information for:**

Horizontal transmission of heat-evolved microalgal symbionts in adult corals

**Supplementary Figures:**


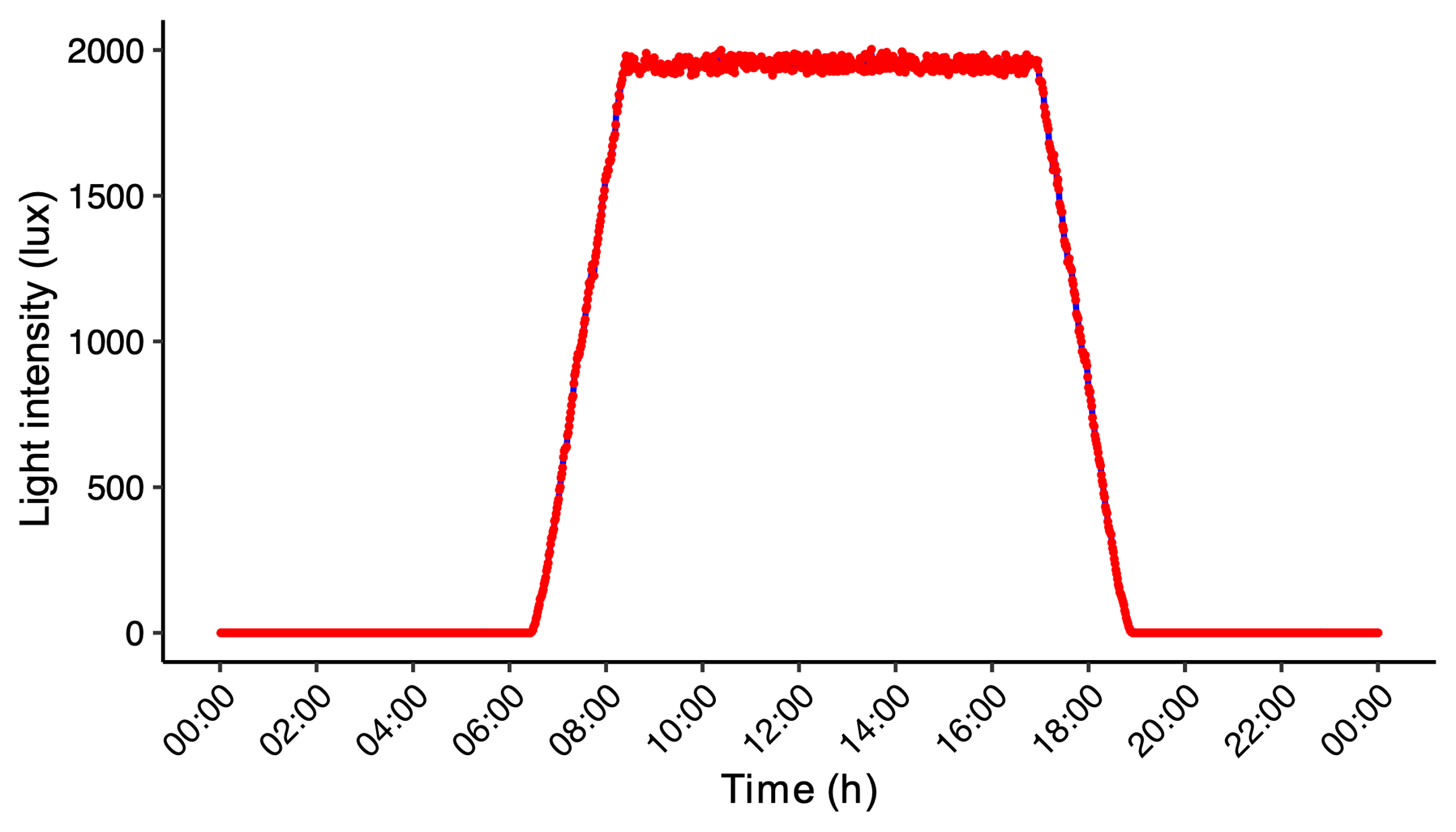
**Fig. S1** Light levels (lux) experienced by *Galaxea fascicularis* over 24 hours throughout the entire experimental period. Ramp time of two hours with peak lux occurring at 08:30 and darkness by 18:30.


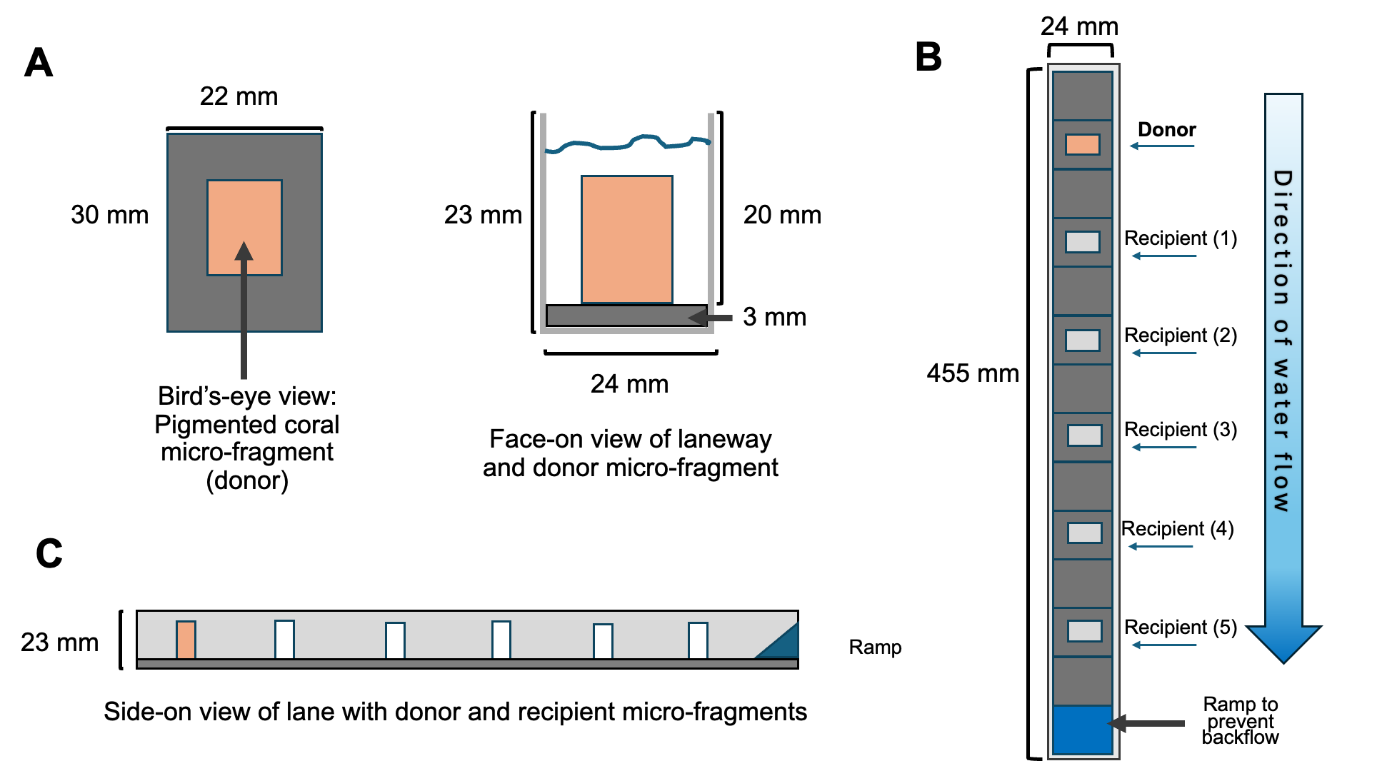
**Fig. S2 Tile and lane specifications of the raceway.** **(A)** Bird’s-eye and face-on view of coral donor on the centre of a PVC tile. Wavy blue line indicates the waterline. **(B)** Bird’s eye view of a singular lane from the raceway showing relative positions of donor and recipient micro fragments. Number next to recipient indicates lane position. **(C)** Side-on view of an individual lane showing relative positions of donor and recipients.


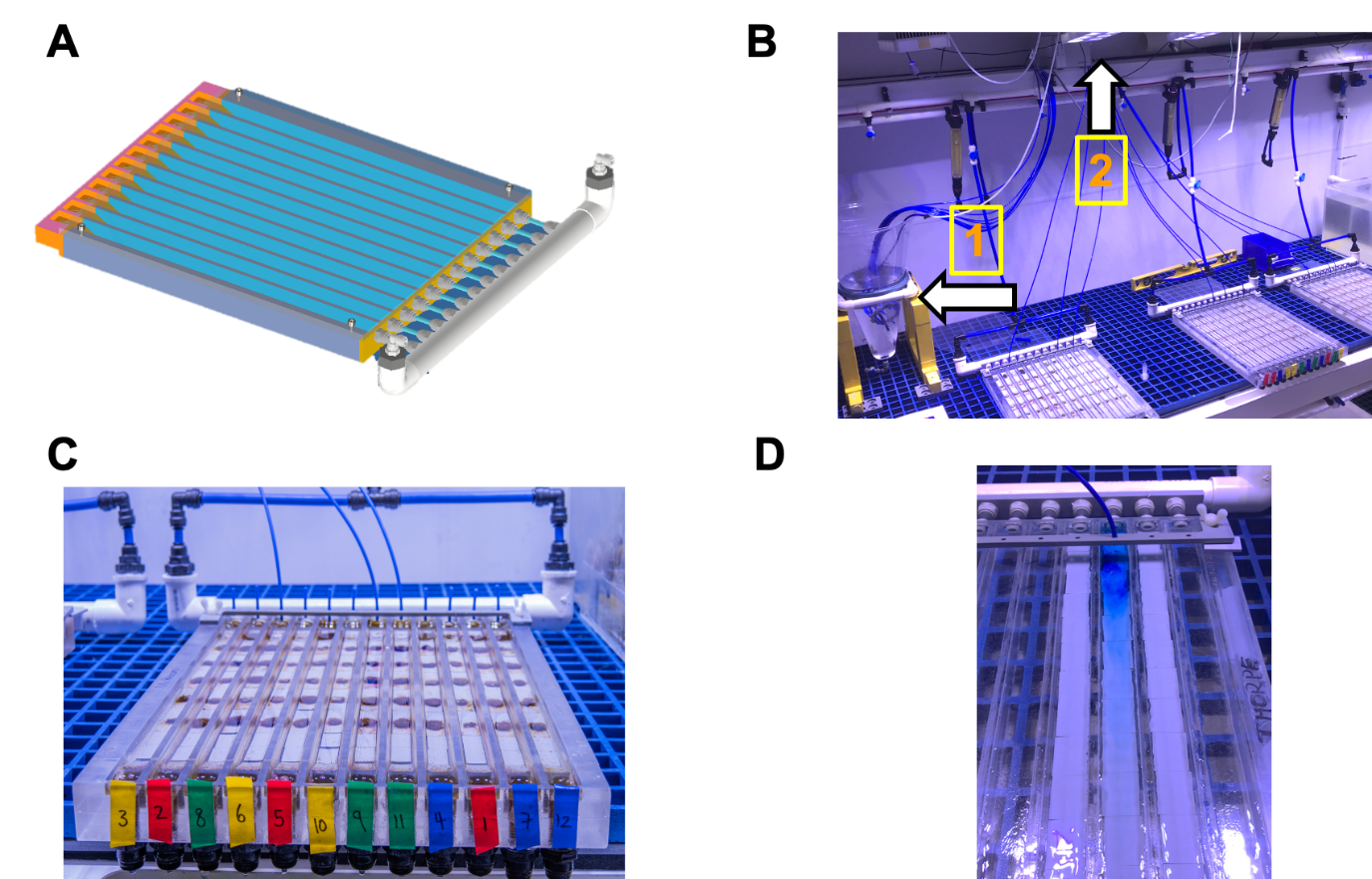
**Fig. S3 Raceway design, experimental set-up, and flow testing. (A)** Graphic representation of the raceway with manifold generated using CAD software (AutoCAD Inventor®, Autodesk Inc., San Rafael, CA, USA). **(B)** Experimental set up including **(1)** symbiont culture cone with gentle aeration to keep SS8 symbionts in suspension and **(2)** peristaltic pump with John Guest tube attachments delivering SS8 cells to culture treatment lanes at a specified volume and cell density. **(C)** The raceway in-operation with coral micro fragments in position and cultured SS8 entering the relevant lanes (green taped lanes). **(D)** Flow visualisation using blue food dye delivered through the symbiont culture cone injection system.

**Fig. S4 Validation of flow speed in the raceway: (A)** Particle tracking of neutrally buoyant fluorescent green polyethylene microspheres (850–1000 μm) within the raceway, visualised under UV light. Microspheres were injected into the raceway manifold to ensure even dispersion across lanes. **(B)** Magnified section showing individual microsphere trajectories, used to quantify flow speed. **(C)** Boxplot depicting flow speed (cm s⁻¹) across each lane. Flow speed was determined by tracking the distance travelled over time of 16 randomly selected microspheres per lane. Tracking was performed on two raceways, with measurements averaged across replicates.


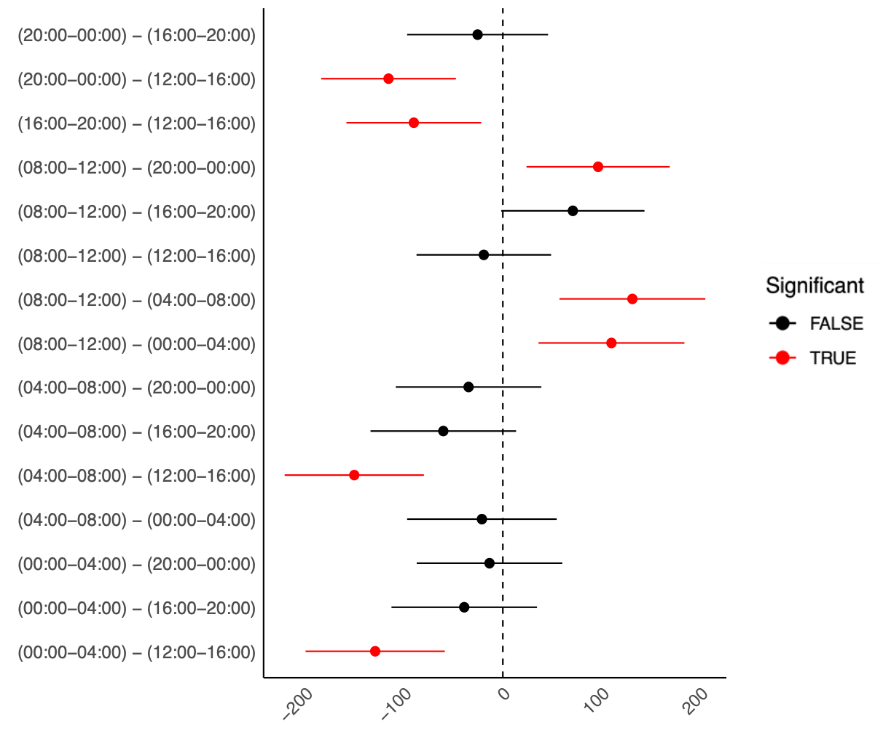


**Fig. S5 Symbiont expulsion rate (hr^-1^ cm^-2^) across timepoints.** Pairwise comparisons of expulsion rates between timepoints, with 95% confidence intervals. Significant comparisons (*P* < 0.05) are shown in red, and non-significant comparisons are in black.


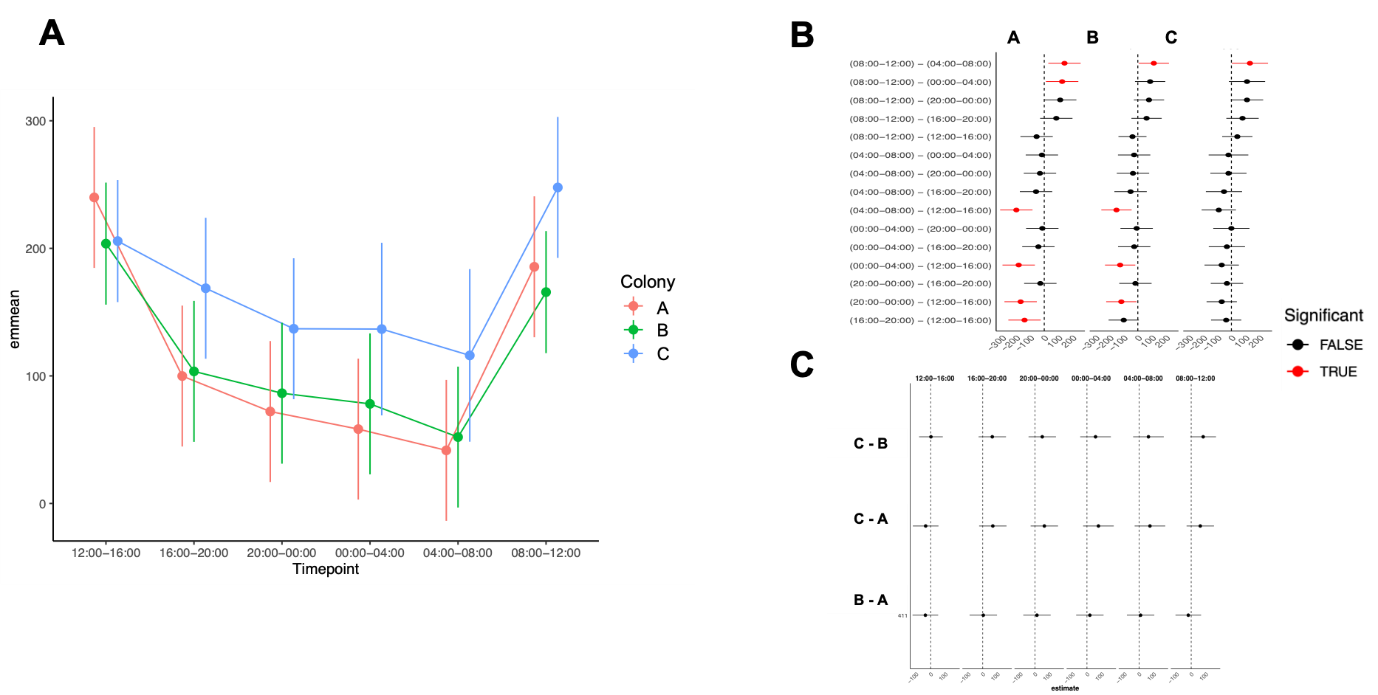
**Fig. S6: Symbiont expulsion rate (hr^-1^ cm^-2^) across incubation timepoint and colony. (A)** Estimated marginal means (emmeans) of symbiont expulsion rate (hr^-1^ cm^-2^) measured across incubation timepoints for three *G. fascicularis* colonies (A, B, C). Points represent mean values, and error bars indicate 95% confidence intervals. **(B)** Pairwise comparisons of symbiont expulsion rate across timepoints between colonies, with 95% confidence intervals. The effect of timepoint on expulsion rate varied between colonies. Diel rhythmicity was more pronounced in colonies A and B, where peak expulsion rates were approximately 5.8× and 3.9× higher than their respective minimums. In contrast, colony C exhibited a smaller fluctuation of 2.1×. **(C)** Pairwise comparisons between colonies across timepoints. No significant differences in overall expulsion rates were detected between colonies at any given timepoint, indicating that although individual fragments exhibited variation in the magnitude of their response, all colonies followed a similar overall diel pattern. Significant comparisons (*P* < 0.05) are shown in red, non-significant comparisons are in black.


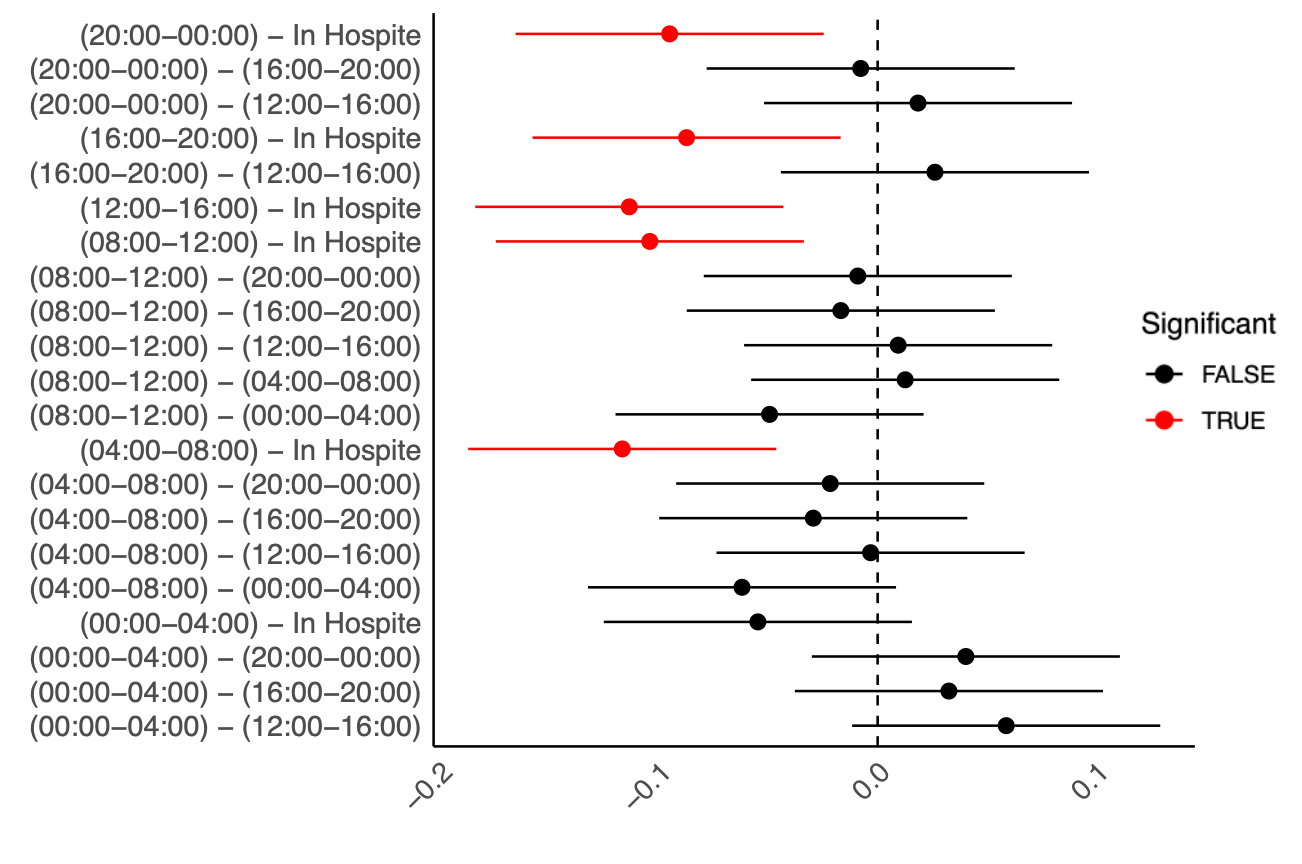


**Fig. S7 Morphologically normal symbionts (%) across timepoints.** Pairwise comparisons of morphologically normal symbionts between timepoints, with 95% confidence intervals. Significant comparisons (*P* < 0.05) are shown in red.


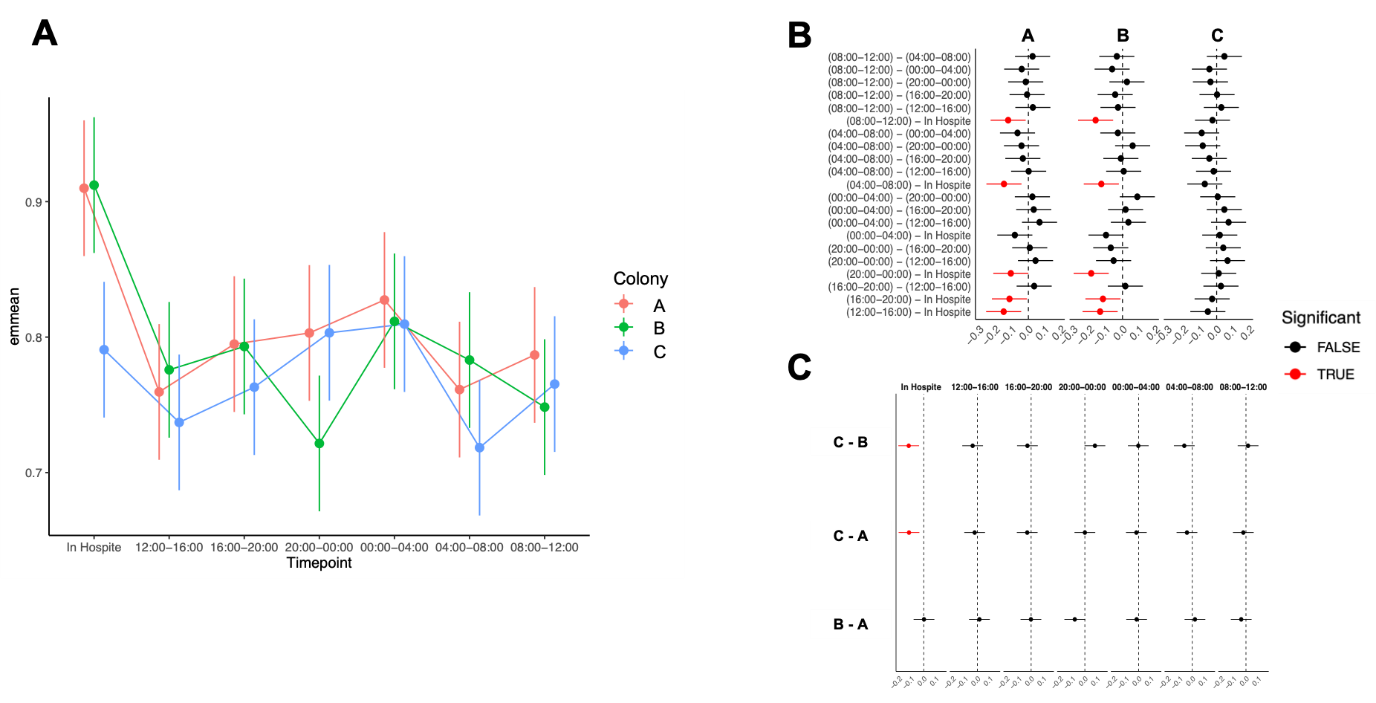
**Fig. S8 Morphologically normal symbionts (%) across timepoints, *in hospite,* and by colony. (A)** Estimated marginal means (emmeans) of morphologically normal symbionts measured across incubation timepoints and *in hospite* for three *G. fascicularis* colonies (A, B, C). Points represent mean values, and error bars indicate 95% confidence intervals. **(B)** Pairwise comparisons of morphologically normal symbionts across timepoints and *in hospite* between colonies, with 95% confidence intervals. Significant colony-level differences were detected within *in hospite* cell populations with pairwise comparisons revealing that colony C had a lower proportion of normal cells (79.1% ± 4.9%) compared to colonies A and B, which together averaged 91.1% ± 2.3%. **(C)** Pairwise comparisons between colonies across timepoints and *in hospite*. No significant colony-level differences in the proportion of normal cells were observed among expelled populations. Significant comparisons (*P* < 0.05) are shown in red.


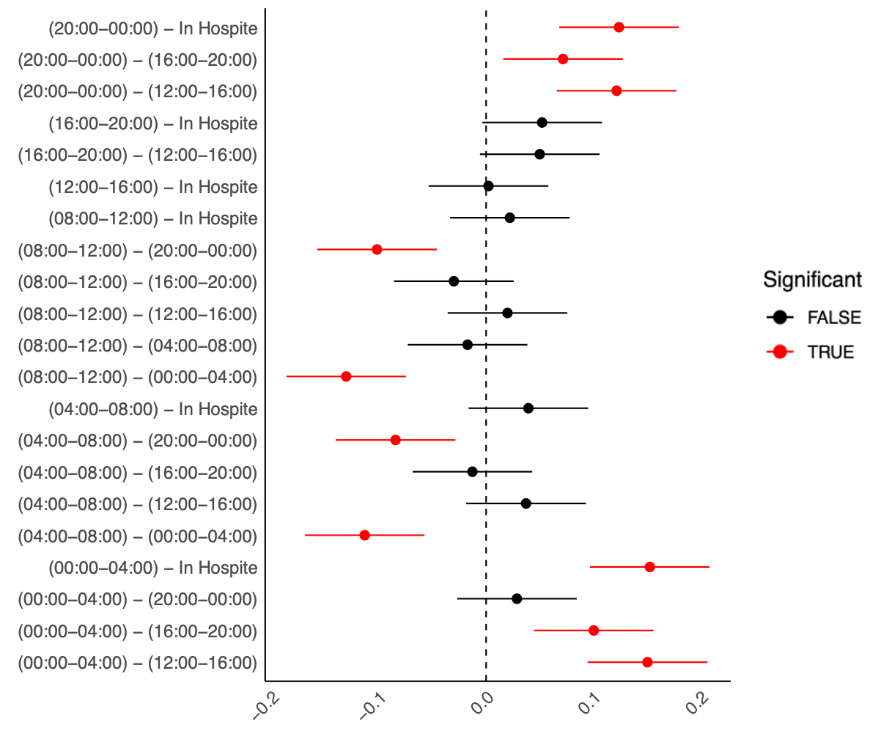


**Fig. S9 Mitotic index across incubation timepoints and *in hospite*.** Pairwise comparisons of mitotic index, with 95% confidence intervals. Significant comparisons (*P* < 0.05) are shown in red.

**Fig. S10 Mitotic index (MI) across incubation timepoints and *in hospite* and by colony.** **(A)** Estimated marginal means (emmeans) of MI measured across incubation timepoints and *in hospite* for three *G. fascicularis* colonies (A, B, C). Points represent mean values, and error bars indicate 95% confidence intervals. **(B)** Pairwise comparisons of MI across timepoints and *in hospite* between colonies, with 95% confidence intervals. The diel cycle had the strongest effect on colony C, which exhibited the largest fluctuation in MI across timepoints. In contrast, colonies A and B followed a similar diel trend but with fewer significant pairwise differences. **(C)** Pairwise comparisons between colonies across timepoints and *in hospite*. Significant comparisons (*P* < 0.05) are shown in red. No significant differences in MI were detected between colonies at any given timepoint, indicating that although the magnitude of the diel effect varied among colonies, the overall temporal pattern was consistent across all colonies.


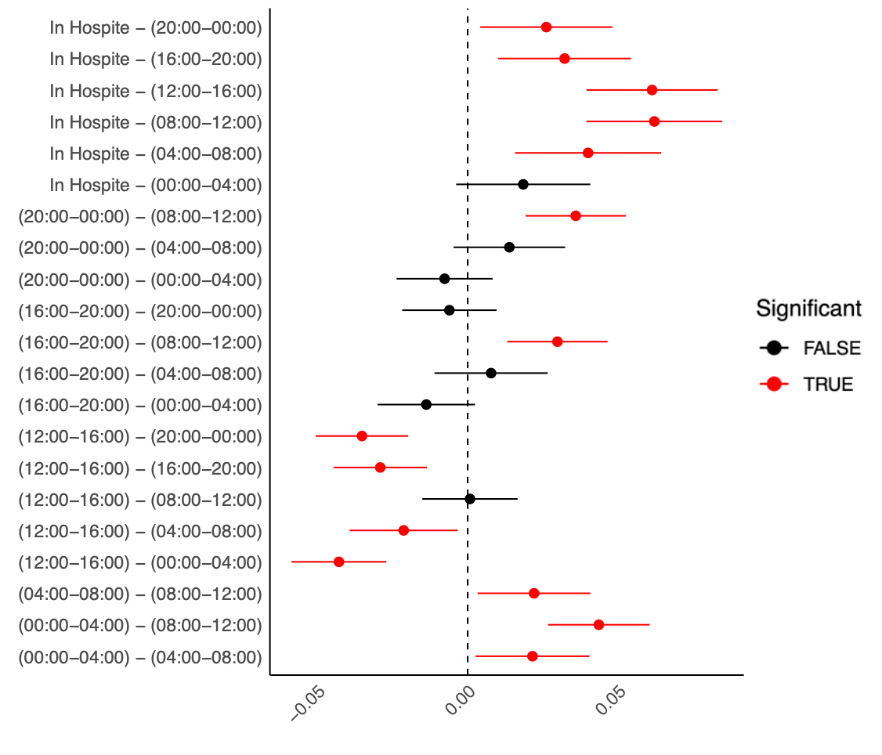


**Fig. S11 Maximum quantum yield of PSII (*F_v_/F_m_***) **across incubation timepoints and *in hospite*.** Pairwise comparisons of *F_v_/F_m_*, with 95% confidence intervals. Significant comparisons (*P* < 0.05) are shown in red.

**Fig. S12 Maximum quantum yield of PSII (*F_v_/F_m_*) across incubation timepoints and *in hospite* and by colony.** **(A)** Estimated marginal means (emmeans) of *F_v_/F_m_* measured across incubation timepoints and *in hospite* for three *G. fascicularis* colonies (A, B, C). Points represent mean values, and error bars indicate 95% confidence intervals. **(B)** Pairwise comparisons of *F_v_/F_m_* across timepoints and *in hospite* between colonies, with 95% confidence intervals. Cells expelled from colonies A and B exhibited more pronounced diel variation in *F_v_/F_m_*, whereas colony C showed a significantly damped response recording the lowest overall values and fewest pairwise differences. *F_v_/F_m_* **(C)** Pairwise comparisons between colonies across timepoints and *in hospite*. Significant comparisons (*P* < 0.05) are shown in red.

**Fig. S13 ITS2 profile and defining intragenomic variant (DIV) community composition of *in hospite* and expelled Symbiodiniaceae.** *In hospite* and expelled ITS2 profiles (top three panels) and DIVs (bottom three panels) from the three *G. fascicularis* colonies (A, B, C) collected over six consecutive 4-hour incubations. Each bar represents expelled or *in hospite* symbionts collected at a specific timepoint.

**Fig. S14 Recipient repigmentation trajectories over time by colony.** Relative colour change of recipient corals over time (Days in Raceway [n]) compared to their initial T0 score, across treatments and colonies. All colonies showed significant increases in pigmentation over the experimental period (*P* < 0.001), with a significant colony:time interaction (*P* < 0.001) indicating different rates of change between colonies. Although there was a trend suggesting that treatment effects varied by both colony and time (treatment:colony:time interaction), this was not statistically significant (*P* = 0.087). Colony C consistently showed the slowest repigmentation rates overall, whereas colonies A and B demonstrated more rapid pigmentation increases over time. Data are shown as mean ± 1 standard deviation.

**Fig. S15 Hellinger transformed ITS2 profile and defining intragenomic variant (DIV) community composition of donors and recipients in the raceway experiment**. Donor and recipient ITS2 type profiles (**A**) and DIVs (**B**) across the four experimental treatments: SS8 donor, SS8 culture (Positive), Homologous donor, and no external symbionts (Negative). Each bar corresponds to an individual recipient sampled at the end of the experiment.

**Fig. S16 SS8 acquisition in the SS8 culture treatment (positive control) varied significantly among colonies.** Boxplots show the proportion of SS8 (%) acquired by recipients from colonies A, B, and C in the cultured SS8 symbiont (positive) treatment. Each point represents an individual recipient. The asterisk (*) denotes a significant comparison (*P* < 0.05, Tukey’s HSD test).

**Supplementary Tables:**

**Table S1**. Symbiont expulsion rate (symbionts expelled hr ^-1^ cm^-2^) over 24 hrs

| **Timepoint** | **Colony A** | **Colony B** | **Colony C** |
| --- | --- | --- | --- |
| 08:00 – 12:00 | 85.53 ± 61.52 | 165.69 ± 63.51 | 247.74 ± 74.79 |
| 12:00 – 16:00 | 239.91 ± 60.48, | 203.7 ± 53.77 | 205.71 ± 21.71 |
| 16:00 – 20:00 | 99.88 ± 93.82 | 103.54 ± 18.75 | 168.71 ± 17.75 |
| 20:00 – 00:00 | 72.08 ± 43.38 | 86.42 ± 23.42 | 136.97 ± 38.65 |
| 00:00 – 04:00 | 58.29 ± 22.08 | 78.08 ± 49.1 | 136.71 ± 2.24 |
| 04:00 – 08:00 | 41.63 ± 30.51 | 52.04 ± 19.89 | 116.07 ± 25.84 |

**Table S2**. Morphologically normal appearing expelled symbionts (%) over 24 hrs.

| **Timepoint** | **Colony A** | **Colony B** | **Colony C** |
| --- | --- | --- | --- |
| *In hospite* | 91.0 ± 2.27 | 91.22 ± 2.22 | 79.1 ± 4.87 |
| 08:00 – 12:00 | 78.78 ± 3.85 | 74.84 ± 4.05 | 76.54 ± 6.99 |
| 12:00 – 16:00 | 75.95 ± 6.82 | 77.59 ± 0.39 | 73.71 ± 5.66 |
| 16:00 – 20:00 | 79.48 ± 4.34 | 79.31 ± 3.87 | 76.31 ± 2.98 |
| 20:00 – 00:00 | 80.30 ± 3.28 | 72.15 ± 6.39 | 80.32 ± 3.53 |
| 00:00 – 04:00 | 82.74 ± 6.17 | 81.16 ± 1.86 | 80.96 ± 5.54 |
| 04:00 – 08:00 | 76.3 ± 2.18 | 78.31 ± 3.72 | 78.31 ± 3.72 |

**Table S3**. Mitotic Index (% dividing cells) of expelled symbionts over 24 hrs

| **Timepoint** | **Colony A** | **Colony B** | **Colony C** |
| --- | --- | --- | --- |
| *In hospite* | 3.83 ± 0.28 | 4.77 ± 1.28 | 8.91 ± 1.59 |
| 08:00 – 12:00 | 8.56 ± 4.66 | 6.63 ±1.36 | 8.96 ± 3.32 |
| 12:00 – 16:00 | 5.81 ± 1.58 | 7.98 ± 2.39 | 4.39 ± 2.91 |
| 16:00 – 20:00 | 7.27 ± 4.26 | 11.84 ± 3.70 | 14.10 ± 2.75 |
| 20:00 – 00:00 | 16.52 ± 3.31 | 15.54 ± 4.10 | 22.60 ± 6.02 |
| 00:00 – 04:00 | 18.42 ± 7.20 | 19.84 ± 2.85 | 25.01 ± 5.84 |
| 00:40 – 00:00 | 10.73 ± 4.815 | 8.73 ± 5.56 | 9.88 ± 2.24 |

**Table S4**. Maximum quantum yield of PSII (*F_v_/F_m_*) of expelled symbionts over 24 hrs

| **Timepoint** | **Colony A** | **Colony B** | **Colony C** |
| --- | --- | --- | --- |
| *In hospite* | 0.26 ± 0.07 | 0.23 ± 0.11 | 0.15 ± 0.09 |
| 08:00 – 12:00 | 0.17 ± 0.07 | 0.17 ± 0.07 | 0.14 ± 0.08 |
| 12:00 – 16:00 | 0.16 ± 0.06 | 0.17 ± 0.07 | 0.16 ± 0.07 |
| 16:00 – 20:00 | 0.19 ± 0.1 | 0.21 ± 0.08 | 0.17 ± 0.08 |
| 20:00 – 00:00 | 0.23 ± 0.06 | 0.22 ± 0.06 | 0.15 ± 0.06 |
| 00:00 – 04:00 | 0.22 ± 0.08 | 0.21 ± 0.07 | 0.18 ± 0.08 |
| 04:00 – 08:00 | 0.20 ± 0.08 | 0.20 ± 0.08 | 0.15 ± 0.1 |

**Table S5.** Recipient mortality in the raceway

| **Treatment** | **Colony A** | **Colony B** | **Colony C** |
| --- | --- | --- | --- |
| SS8 Donor | 0/16 | 0/11 | 3/18 (16.7%) |
| Hom Donor | 0/17 | 0/16 | 3/12 (20%) |
| SS8 Culture | 0/16 | 0/14 | 2/15 (13.3%) |
| No external symbionts | 0/11 | 0/19 | 1/15 (6.7%) |

**Table S6**: Type III ANOVA results for repigmentation trajectories of raceway recipients. Analysis conducted using linear mixed-effects model with compound symmetry correlation structure. Response variable was Box-Cox transformed (λ = 0.22) to improve normality.

| **Source** | **Df** | **χ²** | **Pr(> χ^2^)** |
| --- | --- | --- | --- |
| Treatment | 3 | 1.8 | 0.615 |
| Time | 1 | 447.25 | < 0.001 *** |
| Colony | 2 | 5.30 | 0.071 |
| Treatment:Time | 3 | 6.42 | 0.093 |
| Treatment:Colony | 6 | 6.60 | 0.359 |
| Time:Colony | 2 | 15.91 | < 0.001 *** |
| Treatment:Time:Colony | 6 | 11.04 | 0.087 |

**Table S7** One-way ANOVA testing for differences in SS8 acquisition among recipient colonies within the SS8 culture treatment (positive control)

| **Source** | **Df** | **Sum Sq** | **Mean Sq** |  | **Pr(>F)** |
| --- | --- | --- | --- | --- | --- |
| Colony | 2 | 0. 0005299 | 3.528 |  | 0.0416 * |
| Residuals | 31 | 0.0023279 |  |  |  |

**Supplementary Methods:**

*S1: Collection and maintenance of corals*

Three colonies *of Galaxea fascicularis* (herein referred to as A, B and C) were collected from Davies Reef in the central region of the Great Barrier Reef on October 29, 2021, at depths of 5-10 m (GBRMPA collection permit number: G12/35236.1). Corals were transported to the National Sea Simulator at the Australian Institute of Marine Science (AIMS), Townsville, Australia, and acclimated in aquarium facilities for two weeks. Following acclimation, corals were cut into 4-5 polyp fragments using a diamond blade band saw (Gryphon C-40 Bandsaw) and affixed to aragonite plugs (Frag Plugs Aragonite Large; OW100LCFP, quasonic, Wauchope, Australia) using super glue. Fragments were left to recover in 2 µm filtered seawater (FSW) for four weeks in 50 L experimental tanks with a 5 L per hour turnover rate and an 11-hour photoperiod (6:50-17:50) achieved using Hydra 64HD lights (Aqua Illumination, Bethlehem, PA, USA). Light intensities ranged from 92-180 µmol m^-2^ s^-1^. Corals were fed daily with Artemia nauplii (0.5 nauplii/ml). Filamentous algae and biofilm were removed from the aragonite plugs once per week, and water temperature was maintained at 26.7-27°C. Air stones were used for water movement and aeration.

*S2: Chemical bleaching and reinoculation*

A pilot study was conducted to evaluate the bleaching efficiency of different menthol concentrations (0.19 and 0.39 mM) (M2772, Sigma-Aldrich, St. Luis, MO, USA), both alone and in combination with DCMU (50 µM) (3-(3,4-dichlorophenyl)-1,1-dimethylurea (DCMU; D2425, Sigma-Aldrich) 3-(3,4-dichlorophenyl)-1,1-dimethylurea (DCMU; D2425, Sigma-Aldrich)). The treatment that achieved the greatest removal of native *Symbiodiniaceae* **and** simultaneously minimised coral mortality (0.39 mM menthol without DCMU) was selected for this study.

*G. fascicularis* fragments were incubated in 0.39 mM menthol-spiked FSW for 8 hours (08:30-16:30) for four consecutive days with complete seawater replacement at the end of each incubation. This was followed by a three-day recovery period, together constituting one round. This schedule was repeated for two rounds or until corals appeared visibly bleached, assessed using a high-power stereomicroscope. No feeding occurred during the chemical bleaching or recovery period. Reinoculation involved submerging corals in gently aerated FSW containing cultured SCF055 at a final concentration of 1,000 cells mL⁻¹. Cultures were added at approximately 10:00 h coinciding with the symbiont's peak motile period. Corals were returned to normal flow by 16:40 h, constituting one day of reinoculation. This process was repeated for seven days, followed by an eight-week repigmentation period. Repigmentation was monitored via weekly analysis of colour reflectance and biweekly iPAM for photochemical efficiency. Tissue samples were taken for ITS2 community composition analysis to confirm the presence of SCF055.01.08 (see below for details on ITS2 library preparation).

*S3: Recovery scoring*

To score visual pigmentation scoring, calibration curves were generated using colour standards with known RGB (red, green, blue) brightness values. Pigmentation scores for each coral were then derived from the average brightness value, allowing for standardised quantification of repigmentation. The dark-adapted maximum quantum yield of photosystem II (*F_v_/F_m_*) of corals was measured using the Walz Imaging Pulse Amplitude Modulation (I-PAM) Fluorometer driven by the software ImagingWin. Imaging-PAM parameters were set as follows: light intensity = 4, gain = 3, frequency = 4, damping = 2, actinic light = 2. Data were collected via ImagingWin software (V2.32 FW Multi RGB; Walz GmbH, Effeltrich, Germany). The corals were dark-adapted for 15 min prior to measurement.

*S4: 3D photogrammetry*

All expulsion counts were normalised to the tissue surface area of the corresponding *Galaxea fascicularis* donor, measured using photogrammetry as described by Figueira et al. (2015). Briefly, ~ 40 images of each coral colony were taken while positioned on a PVC holder with Agisoft markers in focus. Photos were captured from two angles to ensure complete surface coverage. Image processing was conducted using Agisoft Metashape Professional, involving photo alignment, dense cloud generation, mesh creation, texture application, scaling, removal of unwanted areas, and surface area measurement.

*S5 Maximum quantum yield of photosystem II of expelled symbionts*

The maximum quantum yield of photosystem II (*F_v_/F_m_*) in expelled symbionts was measured using the second half of the polycarbonate filter. To maintain cell hydration during visualisation, 20 µl of FSW was pipetted onto the halved filter before it was mounted on a glass microscope slide with a coverslip. Measurements were performed on randomly selected individual cells using a Walz Microscopy Pulse Amplitude Modulated (Microscopy iPAM) chlorophyll fluorescence imaging fluorometer. After 15 minutes low-light-adaptation in a humidity chamber, minimum fluorescence (F_O_) was recorded before application of a saturating pulse of light (settings: saturating intensity = 9, saturating width = 14 x 60 ms, actinic intensity = 6, gain = 4, damping = 2) to determine maximum fluorescence (F_m_). Between 20 and 118 individual symbionts were randomly measured per *G. fascicularis* genotype at each timepoint. Measurements were conducted at 20 x magnification, using a 470 nm blue Zeiss LED module for fluorescence excitation, and data were collected via ImagingWin software (V2.32 FW Multi RGB; Walz GmbH, Effeltrich, Germany). Only cells with an F_O_ > 0.1 were included in the analysis.

*S6: DNA extraction, PCR amplification and library preparation*

*DNA extraction*

DNA extractions followed the methodology of Wilson et al. (2002). Briefly, expelled symbionts (as described above) collected on 4 µm polycarbonate filters or coral tissue samples (~2 mm³, see below) were lysed and digested using bead-beating (Sigma G1152 - 100G, 710-1180 µm), lysozyme (67.5 µg/mL), and proteinase K (535 µg/mL). The resulting homogenate was incubated at 65°C for 90 minutes. Following incubation, 250 µL of potassium acetate (KOAc) was added per 1 mL of homogenate. Samples were thoroughly mixed, incubated on ice for 30 minutes, and centrifuged at 25,000×g for 15 minutes at 4°C. The supernatant was then carefully decanted, and DNA precipitation was achieved by adding 0.8 volumes of isopropanol. Genomic DNA was collected by centrifugation at 25,000×g for 12 minutes at room temperature, then washed with 200 µL of 70% ethanol. The supernatant was discarded, and the DNA pellet was air-dried for 20–30 minutes. Finally, the DNA pellet was resuspended in approximately 35 µL of Milli-Q water and stored at 4°C.

*Amplification*
PCR amplification of the Internal Transcribed Spacer 2 (ITS2) region was performed in triplicate using the SYM_VAR_5.8S2 (forward: 5’-GTGACCTATGAACTCAGGAGTCGAATTGCAGAACTCCGTGAACC-3’) and SYM_VAR_REV (reverse: 5’-CTGAGACTTGCACATCGCAGCCGGGTTCWCTTGTYTGACTTCATGC-3’) primers developed by Hume *et al*. 2018 [10] with Illumina adapters underlined. The PCR reactions were set up with 30 µl of Taq PCR Master Mix (201445, Qiagen, Hilden, Germany), 1 µl of 1:10 diluted DNA template, 1.5 µl of forward and reverse primer (10 µM working solution), and 24 µl of Milli-Q ultrapure water. The optimised thermocycling protocol consisted of an initial denaturation step at 95.0°C for 15 min, followed by 18 cycles of denaturation at 95°C for 15 s, annealing at 56°C, and extension at 72°C for 30 s each, with a final extension step at 72°C for 7 min. The triplicate PCR reactions then underwent an indexing PCR reaction using the Nextera XT Library Prep kit (Illumina) with a dual indexing strategy.

Indexed triplicate PCR amplicons were then pooled and cleaned using AMPure XP beads following the manufacturer’s protocol. Product quantity and size were visualized on 1% agarose 1 x TAE agarose gel.

*S7:* *SymPortal Analysis*

Demultiplexed raw sequences were submitted to SymPortal for ITS2 profiling. SymPortal identifies ITS2 profiles based on recurring assemblages of intragenomic variants across samples, representing putative Symbiodiniaceae taxa. Replicates with a single defining intragenomic variant (DIV) were excluded due to insufficient sequence depth. DNA sequence reads ≤10 were removed from all samples to eliminate background contamination. Additionally, sequences detected in DNA extraction negatives were removed from the corresponding samples extracted on the same day. Sequencing data was downloaded from SymPortal and analysed in R under version 4.2.0 (R Core Team, 2022). Prior to analysis, samples with missing status indicators, missing photo IDs, or those marked as dead were removed from the dataset

*S8: Flow validation*

To ensure consistent flow speed and pattern across all lanes and raceways, neutrally buoyant fluorescent green polyethylene microspheres (850–1000 μm; Cospheric LLC, Santa Barbara, CA, USA) were used as flow tracers. Microspheres were treated with a biocompatible surfactant (Tween 20) to achieve neutral buoyancy and visualized under UV light. Flow was recorded from above using a high-resolution camera (UI-3180CP Rev. 2.1; IDS GmbH, Obersulm, Germany) at velocities ranging from 0.5 to 10 cm s⁻¹. Microspheres were injected through the raceway manifold to ensure approximately equal distribution of microspheres across lanes.

Videos were processed in ImageJ (Rasband, 1997) using the Flowtrace algorithm (Gilpin et al., 2017) to map time-varying flow fields and calculate microsphere velocity (n = 16 per lane). To maintain comparable flow conditions across treatments, coral skeletons of similar size to donors were bleached and placed in lanes lacking living coral donors (positive and negative controls)

*S9: G. fascicularis recipient generation for the raceway*

To generate coral recipients for the raceway, *G. fascicularis* colonies (A, B, C) were sawn into, 4-5 polyp micro fragments using a diamond blade band saw (Gryphon C-40 Bandsaw). Micro fragments were allowed to heal for three weeks to permit tissue growth over sections of exposed skeleton before being chemically bleached (see S2). Bleached micro fragments were then attached to the centre of a PVC tile (30 x 22 x 3 mm) using Gorilla Super Glue and randomly distributed across the raceway. Each lane contained five recipients, separated by two empty PVC tiles (~ 65 mm between recipients). Two additional PVC tiles and a chock were placed downstream of the last recipient.

*S10: Cone calculations for cultured SS8 delivery*

Symbiont density of batch SS8 cultures were calculated using a haemocytometer. Briefly, three x 10 µL aliquots were taken and then diluted 1:10 with FSW (due to high cell densities) to enable accurate counting. To achieve a continuous six-hours of SS8 dosing at a concentration of 10,000 cells per mL per lane, the total number of symbionts required was calculated as ~ 32,400,000 cells per day. The corresponding volume of culture was then extracted, added to the dosing cone, and diluted with FSW to achieve the desired 10,000 cells per mL. A peristaltic pump was set-up to dispense 1 mL of culture from the cone per minute to the relevant treatment lanes through 4mm OD x 2.5mm John Guest LLDPE (linear low density polyethylene pipe) tubing (see Fig S2B, C). This equates to 3,600,000 symbionts per lane per day over six hours of dosing (9:30 – 15:30). Flow speed in the raceway was set to approximately 1.3 cm s^-1^ (see Fig S3). Assuming symbionts remain buoyant and do not settle on the tiles, cultured symbionts would thus exist in the lane for approximately 28 seconds before exiting the raceway down the outlet. With approximately 10,000 cells entering the lane per minute, this equates to a symbiont density of approximately 45 cells per mL within a lane (total volume: 218.5 mL) at any given time.
